# Laryngeal Leiomyosarcoma, A Case Report and Review of Articles

**Published:** 2013-10

**Authors:** Ehsan Khadivi, Mohammad Hossein Taziky, Amir Hossein Jafarian, Msoud Nasseri Sadr

**Affiliations:** 1 *Department of Otorhinolarngology, Mashhad University of Medical Sciences, Mashhad, Iran.*; 2 *Department of Otorhinolarngology, Gorgan University of Medical Sciences, Gorgan, Iran.*; 3 *Department of Pathology, Mashhad University of Medical Sciences, Mashhad, Iran.*

**Keywords:** Larynx, Leiomyosarcoma, Mesodermal neoplasms

## Abstract

**Introduction:**

Laryngeal leiomysarcoma is an extremely rare malignancy originating from smooth muscle cells. Its rarity is due to the fact that only less than 50 cases of pure laryngeal leiomyosarcoma and less than 10 cases of hypopharyngeal leiomyosarcaoma have been reported in modern medical literature. Even though the clinical presentation mimics that of a laryngeal carcinoma forming the major bulk of the laryngeal malignancies, the difference in management, warrants an accurate diagnosis.

**Case Report:**

We reported a case of this very rare malignancy presenting in the supraglottic region by highlighting the clinical features, histological and radiological diagnosis and management of this extremely rare malignant entity.

**Conclusion:**

An accurate histological diagnosis may be difficult; but supplementing by electron microscopy and immunohistochemical staining, the diagnosis can be reached certainly.

## Introduction

Malignant mesodermal neoplasms of the larynx are very rare: accounting for less than 1% of all malignant laryngeal tumors (1). Leimyosarcomas of head and neck constitutes 3% of all Leimyosarcomas (2). Of these head and neck Leimyosarcomas; only less than 50 cases originates from laryngeal region and less than 10 cases from the hypopharyngeal region (3,4). This extremely rare malignancy which mimics a laryngeal carcinoma in its clinical presentation and imaging demands accurate diagnosis using immunehisto- chemical modalities due to the differences in the management. The rarity of this malignancy had led to uncertainty with regard to the accurate line of management and prognostic information.

## Case Report

A 49 year-old male represented severe respiratory distress, odynophagia and dysphagia which were associated with biphasic stridor. The respiratory distress had begun one week ago. No history of smoking or alcohol was observed.

**Fig1:**
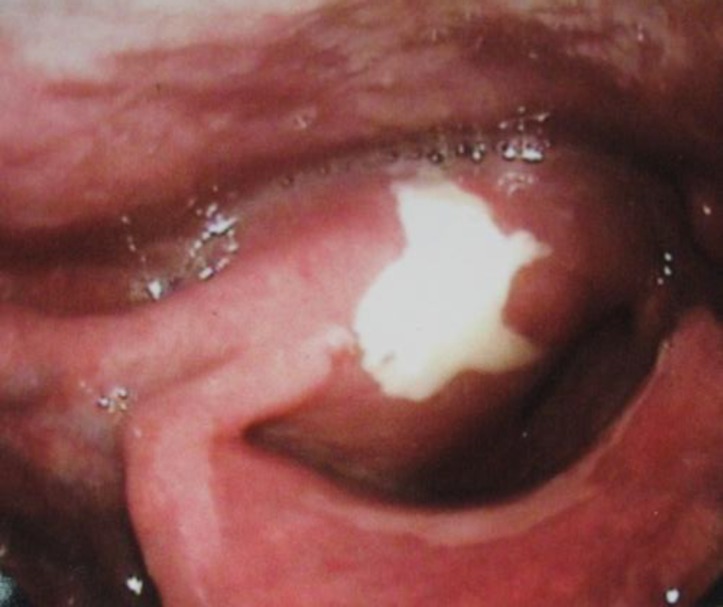
Macroscopic feature of laryngeal leiomyosarcoma

Emergent tracheostomy was done. Direct layngoscopy under GA showed a proliferative growth in the supraglottic region extending towards the right side in the aryepiglottic area. There was no damage on growth. Right TVC and right FVC and ant. commissure were tumoral. Left TVC was mobile (Fig. 1). 

The medial wall of right pyriform sinus was tumoral but the apex was normal. The other subunits of hypopharynx were normal. The biopsy of tumor was reported to be granulation tissue.

**Fig 2:**
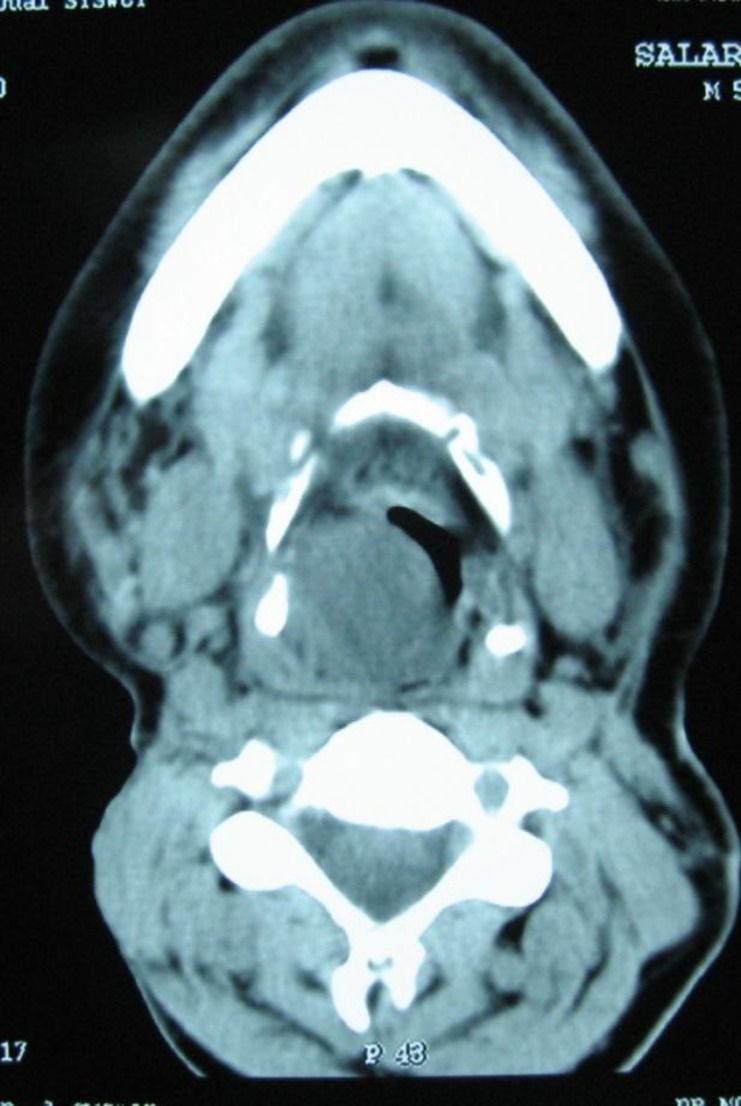
Axial CT scan of larynx

CT scan of the soft tissue of neck showed an irregular opacity in the sub-mucosal region of right supraglottis in central necrosis. The opacity projected into the airway and caused significant obstruction and invaded tumor thyroid cartilage (Fig.2).

In the past medical history of the patient, he has had papillary cell carcinoma of thyroid 37 years ago which underwent right hemithyro-idectomy and again 30 years ago he has had suffered left hemithyriodectomy and bilateral neck dissection and external RT. After the second surgery, because of aspiration and dysphonia, injection thyroplasty type 1 was done by Teflon on the right side.

Due to the positive history for Teflon injection, it was assumed that granulation tissue on biopsy is Teflon granulation. The signs and symptoms gradually became more severe and second biopsy was done.

**Fig 3:**
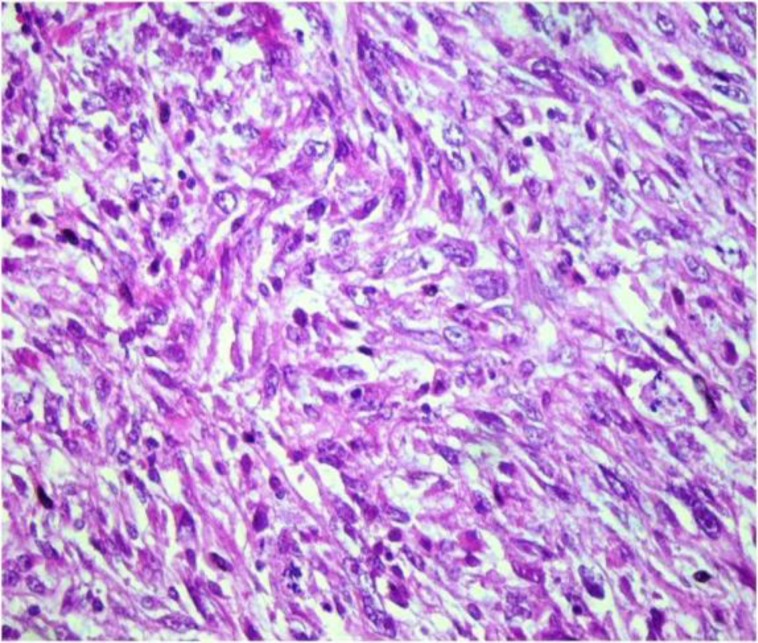
fasicles of eosinophilic spindle cells, with vesicular, cigar shaped nuclei (H&E ×400).

The second biopsy suggested a malignant mesenchymal tumor. Histological examina- tion showed fascicles of eosinophilic spindle cells, and vesicular texture which is a cigar shaped nuclei with moderate atypia and 10 mitotic figures per high power field (Fig.3).

**Fig4:**
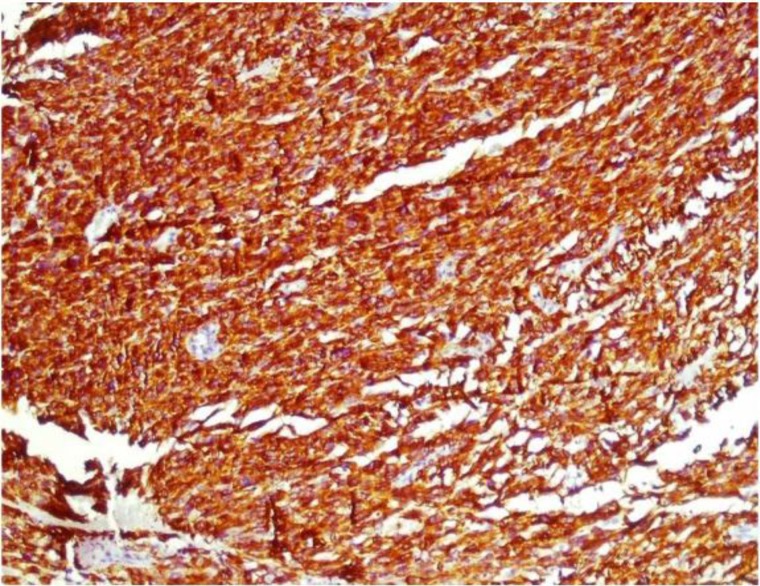
Immunohistochemical positive reactivity for Desmin (×100).

The immunohistochemistry of the specimen was positive for alpha smooth muscle actin, desmin (Fig.4),vimentin and negative for S100 and pancytokeratin was consistent with the diagnosis of lieomyosarcoma of larynx. 

The patient was then investigated to rule out the possibility of disseminations, which all were negative. Total laryngectomy was done postoperatively; the patient was treated by Adjuvant Radiochemotherapy and followed up.

## Discussion

Malignancies of the larynx account for 1-5% of malignancies diagnosed annually. Majority of these tumors are squamous cell carci- nomas. Mesenchymal tissue malignancies or sarcomas form a very insignificant proportion of laryngeal malignancies; accounting roughly 1% (4). Leiomyosarcoma of the larynx was first reported by Jackson in 1939(5).

Leiomyosarcoma originates from the smooth muscles tissue. It occurs in the region where smooth muscles are in plenty; namely uterus, the gastrointestinal tract and the retroperitoneum. Head and neck leiomyosar- comas account only for 3% of all leiomyosarcomas (6). The low Leiomyosar- coma incidence in the head and neck region is explained as a result of smooth muscle scarcity in the head and neck, which is limited to erector pili muscles, vessele walls and the esophagus. The common sites of head and neck leiomyosarcomas are paranasal sinuses, scalp and cervical esophagus (4).

Majority of the cases related to the larynx leiomyosarcomas originates in the supraglo- ttic or glottic area (7). Marioni et al in 2000 (8); in his review of 31 cases, reported the incidences in laryngeal subsites. The authors noted that the incidence of each sites was: glottis (48%), supraglottis (32%),supraglottis–glottis (6.5%), subglottis (6.5%), supraglottis–glottis-subglottis (3.5%) and glottis- subglottis (3.5%) (8). 

In our patient, the tumor also showed an extension in the hypopharynx (medial wall of piryform sinus), which is an even rarer clinical feature. Extensive review of the modern literature showed that there were only less than 10 cases of hypopharyngeal leiomyosarcoma reported; adding to the rarity of the present case (2).

 The etiological factors which play in the genesis of this rare malignancy are still unclear unlike the squamous cell carcinomas of the larynx in which smoking and alcohol were important etiological components, their role in leiomyosarcoma larynx is still uncertain. An aberrant differentiation of mesenchyma is postulated as a factor, suggested by the factor that a leiomyosarcoma can occur in an area where smooth muscles are in rarity, namely the head and neck, which may be possible in our patient (9). Another argument which is stated in medical literature as an etiological factor is a post operative healing process deviating from its normalcy; which might be the cause here as the patient has a history of surgical interventions in the past (10). There have been reports of Ebstein- Bar virus genome associated with this rare malignancy; especially in immunosuppressed patients (11). 

The diagnosis of a leiomyosarcoma larynx is difficult to appreciate clinically as they are clinically indistinguishable from a laryngeal carcinoma. Hoarseness is a common complaint. But this non-specific complaint occurs both in benign as well as malignant laryngeal growths (4). Laryngeal leiomyosar- coma presented as an obstructive feature has been reported in medical literature. Tewary et al had reported a case which required an emergency laryngectomy for airway presentation (12). The patients are reported to be managed by an emergent tracheotomy (13).These patients had obstructive complaints 

Head and neck leiomyosarcomas rarely are presented by lymph node metastasis. In our case, there was no lymphadenopathy.

The role of conventional microscopy is limited for making a morphological diagnosis of laryngeal leiomyosarcoma. When the findings are supplemented by immunohisto- chemistry and ultrastructural investigations, the diagnosis of this rare tumor is made certainly. Histopatho- logically, laryngeal leiomyosarcoma is characterized by prominent interlacing bundles and fascicular arrangement of spindle-shaped cells with cigar-shaped, blunt–ended nuclei and eosinophilic cytoplasm.

Other spindle cell tumors include rhabdomyosarcoma, melanoma, schwanoma, malignant fibrous histiocytoma and sarcomatoid carcinoma. The advent of newer monoclonal antibody techniques aiding an immune- histochemical diagnosis has made the diagnosis easier. Leiomyosarcomas are usually positive for alpha smooth muscle actin and negative for cytokeratins and epithelial membrane antigens. In our case, the tumor was positive for alpha smooth muscle actin, desmin and vimentin and negative for pancytokeratin and S-100 antigens.

In 2005, Marioni et al. (8) stressed the diagnostic role played by immunohisto- chemistry in the diagnosis of this rare malignancy. The authors stressed that, even though this tests allow a reliable diagnosis, it can produce ambiguous or inconclusive results when the tumor cells lack specific immunohistochemical reactivity. In this rare scenario, the authors suggest the diagnosis to be substantiated by electron microscopy because in addition to academic interest, any wrong assumption might lead to inappropriate clinical management (14).

Radiological investigations like CT and magnetic resonance imaging play a critical role to determine the extent of the primary surgery planning as well as delivering the information regarding the lymph node status.

The rarity of this tumor has resulted in the lack of any conclusions regarding the standard modality of management.

Abbas et al in 2005(1) in their review suggested that the first line of management be resection of the whole tumor with wide surgical margins with curative intent.

In certain cases, endolaryngeal resection or partial laryngectomy has been tried to preserve functionality in early disease status. Radical neck dissection is reported to be reserved in case of obvious cervical lymphadenopathy. The rate of local recurrence following surgical excision ranges from 35-50% (3). Our patient was treated by laryngectomy for complete removal of the disease with a curative intent.

The role of radiotherapy is to be an adjuvant therapeutic modality. Radiation can also have a role in recurrence or in residual disease, but as a primary treatment modality, radiotherapy is less effective (15). Our patient received adjuvant external beam radiotherapy in parallel opposed fields. Chemotherapy has also a limited role in leiomyosarcomas of the larynx.

The scarcity of cases has caused the indecisiveness for chalking out the prognosis of this rare tumor. Literature search has shown that head and neck leiomyosarcomas generally have a survival of 35-50 % (4). Medical literature review showed that the longest period of postoperative disease is 11 years. A long period of follow up is mandatory since the risk of recurrence persist long time after treatment (3).

## Conclusion

With the advent of new immunohisto- chemical techniques, the diagnosis of leiomyosarcomas of the larynx can be picked out from the list of differential diagnosis of spindle cell tumors of the larynx. Such modalities should be freely used for diagnosis in case of the wide difference in the treatment methods for giving the patient a better cure and quality of life.
